# Specialty-based differences in osteoporosis management in Türkiye: a national cross-sectional survey

**DOI:** 10.1007/s11657-026-01717-6

**Published:** 2026-05-21

**Authors:** Muhammed Yasir Altunışık, Zekeriya Keskin, Burak Aydın, Musa Polat

**Affiliations:** 1 Department of Orthopaedics and Traumatology, Faculty of Medicine, Sivas Cumhuriyet University, Sivas, 58140 Turkey; 2 Department of Internal Medicine, Faculty of Medicine,, Sivas Cumhuriyet University, Sivas, 58140 Turkey; 3 Department of Physical Medicine and Rehabilitation, Faculty of Medicine, Sivas Cumhuriyet University, Sivas, 58140 Turkey

**Keywords:** Osteoporosis, Treatment gap, Awareness, Guideline adherence, FRAX, DXA

## Abstract

***Summary*:**

This study showed significant specialty-based differences in osteoporosis management in Türkiye. Physical medicine and rehabilitation (PM&R) physicians demonstrated the highest awareness, whereas orthopedic physicians demonstrated the lowest. Guideline use, rather than seniority, was associated with better awareness, supporting targeted education and wider fracture liaison services (FLS) implementation.

**Purpose:**

This study aimed to compare adherence to current guidelines, clinical approaches, and awareness levels among physicians in internal medicine (IM), PM&R, and orthopedics and traumatology who are actively involved in osteoporosis management in Türkiye.

**Methods:**

A total of 447 physicians from across Türkiye were included (122 IM, 203 PM&R, and 122 orthopedic physicians). A questionnaire was used to evaluate approaches to diagnosis, treatment, and follow-up. An osteoporosis awareness score was calculated as the mean of guideline-based awareness items (Cronbach’s *α* = 0.890). Data were analyzed using the chi-square test, Kruskal-Wallis test, and multivariable linear regression.

**Results:**

Awareness scores differed significantly across specialties (*p* < 0.001). The median (IQR) score was highest in PM&R physicians at 4.00 (3.75–4.25), followed by IM physicians at 3.50 (3.00–4.00) and orthopedic physicians at 2.88 (2.00–3.50). Significant between-specialty differences were also observed in diagnostic evaluation, pre-treatment laboratory assessment, denosumab preference, planning of treatment transition after discontinuation, and follow-up strategies (all *p* < 0.05). In multivariable regression analysis, specialty (*B* = 0.782, *p* < 0.001) and active guideline use (*B* = 0.344, *p* < 0.001) were the strongest independent predictors of awareness score, whereas years of professional experience and academic title were not significant.

**Conclusion:**

Osteoporosis management in Türkiye differs substantially by specialty. Lower concordance with guideline-based care among orthopedics and traumatology physicians may contribute to the treatment gap. Guideline-oriented education should therefore be prioritized over professional seniority, and multidisciplinary FLS should be further expanded.

## Introduction

Osteoporosis is a systemic skeletal disease that increases bone fragility as a result of low bone mass and deterioration of bone microarchitecture, and, particularly through fragility fractures, causes significant morbidity, mortality, and a burden on healthcare services [1]. It is estimated that 1 in 3 women and 1 in 5 men over the age of 50 worldwide will experience an osteoporotic fracture during their lifetime; in Türkiye, the prevalence of osteoporosis among individuals aged 50 years and older is reported to be approximately 25%, and the annual number of hip fractures is expected to increase significantly in the coming years [2–4]. This clinical and societal burden associated with osteoporosis has necessitated moving beyond a narrow framework in which the disease is evaluated solely based on bone mineral density. Therefore, contemporary osteoporosis management requires a comprehensive approach that includes the prediction of fracture risk, identification of individuals at high and very high risk of fracture, timely initiation of appropriate pharmacological treatment, and prevention of secondary fractures [1].

The complexity and chronic nature of osteoporosis management make it difficult to address the disease within the boundaries of a single medical specialty. Diagnostic evaluation, investigation of secondary causes, selection of pharmacological treatment, secondary prevention after fracture, reduction of fall risk, and rehabilitation are often conducted at different clinical touchpoints [2]. A consensus report published in Türkiye states that patients with osteoporosis are mainly managed by specialists in PM&R, endocrinology, rheumatology, IM, orthopedic, and gynecology physicians [2]. Similarly, the FLS model, developed for post-fragility fracture care, also demonstrates that osteoporosis care is essentially multidisciplinary; orthopedic evaluation, fracture risk classification, pharmacological treatment, follow-up, and rehabilitation must be conducted in a coordinated manner [5, 6].

This multidisciplinary structure not only necessitates interdisciplinary collaboration in osteoporosis care but also raises the question of the extent to which diagnosis, risk classification, and treatment decisions can be standardized across different specialties. National and international guidelines serve as important reference points for conducting clinical practice within a common framework in osteoporosis management. However, the existence of these guidelines alone does not guarantee standardized care. International data show significant differences among physicians in terms of knowledge level, guideline use, clinical confidence, and daily practice in osteoporosis care; significant gaps persist, particularly in the interpretation of treatment thresholds, systematic case follow-up, fall risk assessment, and monitoring processes [7–10]. Data from Türkiye are limited, with the only study comparing the clinical approach of different specialties being that of Bakılan et al., which evaluated the awareness of fragility fractures and FLS among orthopedic physicians and neurosurgeons in a single center [11]. In this study, although the relationship between low-energy trauma fractures and osteoporosis is generally accepted, the low awareness of Fracture Risk Assessment Tool (FRAX) and FLS, and the reporting that most patients in the follow-up and treatment of osteoporosis are referred to other specialties—especially to PM&R—indicate that coordinated care models have not been adequately integrated into clinical practice. This situation suggests that there is a need for studies that directly compare how well the main specialties actively involved in osteoporosis management adhere to current guidelines.

Although osteoporosis management is a multidisciplinary field of care, patients in Türkiye do not usually present directly to subspecialty osteoporosis clinics; the initial assessment, treatment decisions, and referral processes are mostly shaped by the Departments of IM, PM&R, and Orthopedics and Traumatology. Therefore, the aim of this study was to comparatively evaluate physicians’ compliance with current guidelines, their clinical approaches, and their level of awareness in these three specialties. Such a comparison can reveal possible differences in practice between specialties, educational needs, and the structural gaps hindering standardized osteoporosis care, thereby contributing to the development of a more consistent and guideline-based care model.

## Methods

### Research design

This study is a descriptive, cross-sectional online survey designed to evaluate physicians’ clinical practices and approaches regarding the diagnosis, treatment, and follow-up of patients with osteoporosis in Türkiye. The study was approved by the Ethics Committee for Health Sciences Research of Sivas Cumhuriyet University (Decision No: 2025-12/28) and conducted in accordance with the principles of the Declaration of Helsinki. Electronic informed consent was obtained from all participants on the entry screen before starting the survey.

### Research population

The target population of the study comprised physicians who were most frequently involved in the clinical care of patients with osteoporosis. The study included research assistants, specialist physicians, and faculty members working in IM, PM&R, and orthopedics and traumatology. These three specialties were chosen because, in Türkiye, patients cannot directly access subspecialty outpatient clinics, and because the initial assessment, treatment decisions, and referral processes in osteoporosis care are largely conducted through these main specialties. Participants were included in the study if they were actively working in one of the three specified specialties and provided affirmative consent to participate, as indicated in the survey. Those who filled out the survey incompletely and four participants who stated that they had not treated or monitored patients with osteoporosis in the past 12 months were excluded from the study. A total of 447 physicians were included in the final analysis.

### Survey development and administration

The survey was administered online using the Google Forms platform. The survey link remained active for 1 month, from January 1 to 31, 2026. The invitation to participate was distributed through professional association groups and messaging applications used by physicians. The survey was completed anonymously by participants based on self-reporting. To minimize the risk of duplicate responses, the “Limit to 1 response” feature on the survey platform was enabled to ensure that each Google account is limited to one response. Incomplete responses were automatically flagged and excluded from the analysis. No incentive was offered for participation, which further reduced the likelihood of coordinated or influenced responses.

The survey form was developed by the research team with reference to current recommendations from the 2025 Turkish Society of Endocrinology and Metabolism guideline, the 2024 National Osteoporosis Guideline Group guideline, and the 2019 European guidance for the diagnosis and management of osteoporosis in postmenopausal women [1, 12, 13]. These guidelines were selected to ensure that the survey items reflected a comprehensive, multidisciplinary standard of care rather than the perspective of any single specialty. Before implementation, the survey was pilot tested with three experienced academics from different specialties. During this process, the clarity, comprehensibility, clinical relevance, and content adequacy of the items were reviewed. The survey was finalized based on the feedback received. Data were collected anonymously and stored in a secure electronic environment accessible only to the research team.

### Survey content and variables

The questionnaire, which takes approximately 5–7 min to complete, consisted of five main sections and 36 questions: demographic and professional characteristics (7 questions), diagnostic approaches and evaluation processes (6 questions), treatment preferences (11 questions), follow-up and monitoring strategies (8 questions), and general awareness questions related to osteoporosis management (4 questions). The survey included both single-response and multiple-response questions. In multiple-response questions, each option was considered a separate variable based on whether it was selected by each participant.

#### Statistical analysis

Since the survey consisted of 36 items, a minimum of 360 participants was targeted by considering the commonly used approach of approximately 10 participants per item in scale- and survey-based research [14]. To further evaluate the adequacy of the final sample size, a post-hoc power analysis was performed using G*Power (version 3.1.9.7). For the primary between-specialty comparison, with a total sample size of 447, three groups, and an approximate observed effect size of *f* = 0.588, the achieved power was > 0.99. In addition, a sensitivity analysis indicated that this sample size was sufficient to detect effect sizes as small as *f* = 0.15 at *α* = 0.05 and a power level of 0.80. For the multivariable linear regression model, a separate post-hoc power analysis (*f*^2^ = 0.621, *α* = 0.05, *n* = 447, predictors = 5) also yielded a power of > 0.99.

All statistical analyses were performed using IBM SPSS Statistics (version 25.0; IBM Corp., Armonk, NY, USA). Descriptive statistics were presented as number (*n*) and percentage (%). Normality of continuous variables was assessed using visual methods (histogram/Q-Q plot) and the Kolmogorov-Smirnov test. For the comparison of non-normally distributed continuous variables, the Kruskal-Wallis test was used; for pairwise comparisons of significant results, the post-hoc Dunn test with Bonferroni correction was preferred. Non-normally distributed continuous variables were presented as median (interquartile range). For the comparison of categorical variables, the Pearson chi-square test was used, and the effect size was reported with Cramer’s V. When more than 20% of the cells had expected values < 5, Monte Carlo exact *p*-values were reported. For multiple-response survey items, the multiple dichotomy method was applied; each option was analyzed as an independent binary variable (selected/not selected). When overall significance was detected between groups, pairwise comparisons of column proportions in the same row were performed using Bonferroni-corrected *Z*-tests to determine specific differences between specialties, and significant differences are indicated with superscript letters (^a^, ^b^, ^c^) in the tables. To display significant differences in the table, the SPSS Custom Tables lettering logic was used, where the letter of the column with the lower proportion appears in the column with the higher proportion (*p* < 0.05).

An “osteoporosis awareness score” was created and used as the dependent variable in the regression analysis. This score was calculated based on four specific questions related to awareness, which included guideline-based practices covered in the survey. Each item was evaluated using a 5-point Likert scale (1 = strongly disagree, 5 = strongly agree). The internal consistency of the scale was assessed using Cronbach’s alpha and was high (*α* = 0.890). The awareness score was obtained by calculating the average of these items, and higher scores were defined as a continuous variable representing a higher level of awareness. To identify the independent determinants of the osteoporosis awareness score, a multivariable linear regression model was established. The independent variables included in the model were selected from factors that showed a significant relationship with the awareness score in univariate analyses (*p* < 0.10) and professional parameters highlighted as clinically important in the literature. The model included the medical specialty (dummy coded with orthopedic physicians as the reference), guideline use, total years of professional experience, academic title, and monthly patient volume. The problem of multicollinearity between variables was checked using the variance inflation factor (VIF); it was determined that all VIF values were < 3.0, and the assumptions were met. The overall explanatory power of the model was reported with the *R*^2^ value, and model significance was assessed using the *F*-test. The level of statistical significance was set at two-sided *p* < 0.05.

## Results

A total of 447 physicians were included in this study. Of these, 122 were IM physicians (27.3%), 203 were PM&R physicians (45.4%), and 122 were orthopedic physicians (27.3%). The majority of participants (57.0%) were at the level of specialist physicians, while 27.2% were residents. Regarding years of professional experience, 71.4% had 5 years or more of experience. No significant difference was found between specialties regarding academic rank (*p* > 0.05). However, statistically significant differences were identified between specialties regarding the type of institution where they worked (*p* = 0.002), monthly patient volume (*p* < 0.001), awareness and use of guidelines (*p* < 0.001), and the presence of a FLS at their institution (*p* < 0.001). In the PM&R group, monthly patient volumes were clustered in higher categories (especially 21–40 and ≥ 41 patients/month), while in the IM and orthopedic groups, the 0–10 patients/month category was reported more frequently. The rate of reporting the presence of an FLS was higher in the PM&R group, whereas in the IM and orthopedic groups, the “I do not know” response was more common. PM&R physicians appeared to have greater awareness and use of guidelines. The proportion of those who consulted and regularly used guidelines when needed was 72% in the PM&R group and 42.6% in the IM group, while it dropped to 26.2% in the orthopedic group. A comparison of demographic data by specialty is presented in Table 1.

**Table 1:** Demographic and professional characteristics of participants

		IM (***n*** = 122)^a^	PM&R (***n*** = 203)^b^	Orthopedics (***n*** = 122)^c^	***p***-value
		*n* (%)	*n* (%)	*n* (%)	
Academic rank	Resident	22 (18.0%)	54 (26.6%)	32 (26.2%)	0.097
	Specialist	82 (67.2%)^b^	106 (52.2%)	67 (54.9%)	
	Assistant professor	9 (7.4%)	9 (4.4%)	9 (7.4%)	
	Associate professor	5 (4.1%)	14 (6.9%)	7 (5.7%)	
	Professor	4 (3.3%)	20 (9.9%)	7 (5.7%)	
Total professional experience	0–5	26 (21.3%)	70 (34.5%)^a^	32 (26.2%)	0.020
	6–10	55 (45.1%)^b^	57 (28.1%)	37 (30.3%)	
	11–15	16 (13.1%)	25 (12.3%)	26 (21.3%)	
	16–20	11 (9.0%)	17 (8.4%)	11 (9.0%)	
	21+	14 (11.5%)	34 (16.7%)	16 (13.1%)	
Current institution type	State hospital	48 (39.3%)	55 (27.1%)	36 (29.5%)	0.002
	Training and research hospital	25 (20.5%)	70 (34.5%)^a,c^	26 (21.3%)	
	University hospital	44 (36.1%)	55 (27.1%)	50 (41.0%)^b^	
	Private hospital	5 (4.1%)	23 (11.3%)	10 (8.2%)	
Monthly patient volume	0–5	49 (40.2%)^b,c^	9 (4.4%)	29 (23.8%)^b^	< 0.001
	6–10	38 (31.1%)^b^	33 (16.3%)	36 (29.5%)^b^	
	11–20	15 (12.3%)	50 (24.6%)^a^	30 (24.6%)^a^	
	21–40	9 (7.4%)	45 (22.2%)^a,c^	14 (11.5%)	
	41+	11 (9.0%)	66 (32.5%)^a,c^	13 (10.7%)	
Guideline awareness and use	Never heard of it	34 (27.9%)^b^	13 (6.4%)	47 (38.5%)^b^	< 0.001
	Aware of the name only	36 (29.5%)	44 (21.7%)	43 (35.2%)^b^	
	Consult it when needed	47 (38.5%)	114 (56.2%)^a,c^	31 (25.4%)	
	Use it regularly	5 (4.1%)	32 (15.8%)^a,c^	1 (0.8%)	
Availability of a fracture liaison service in the institution	Yes	0 (0.0%)	27 (13.3%)^c^	3 (2.5%)	< 0.001
	No	77 (63.1%)	157 (77.3%)^a^	82 (67.2%)	
	Do not know	45 (36.9%)^b^	19 (9.4%)	37 (30.3%)^b^	

A two-sided *p*-value < 0.05 was considered statistically significant

Pairwise comparisons of column proportions within the same row were performed using Bonferroni-adjusted *Z* tests; for significant differences (*p* < 0.05), the letter of the column with the smaller proportion is displayed in the column with the larger proportion

*PM&R*, physical medicine and rehabilitation; *IM*, internal medicine

### Diagnostic and assessment questions

Significant differences were observed between specialties in questions related to the diagnosis and evaluation of osteoporosis. Regarding the indications for requesting dual-energy X-ray absorptiometry (DXA), those who selected > 3 months of glucocorticoid use differed significantly among PM&R (94.1%), IM (69.7%), and orthopedic groups (28.7%) (*p* < 0.001). Those who requested DXA after low-trauma fractures were also significantly higher in the PM&R group (97.0%) than in the IM group (71.3%) and the orthopedic group (70.5%) (*p* < 0.001). Those who never screened for secondary causes were more common in the orthopedic group (21.3%), while those who conducted screening if there were risk factors were more frequent in the IM (76.2%) and PM&R (71.4%) groups (*p* < 0.001). The specialty with the highest rate of FRAX use as a Fracture Risk Assessment Tool was the PM&R group (75.4%), while the orthopedic group had the lowest rate (11.5%). The proportion of those who did not routinely perform fall risk assessments was higher in the orthopedic (37.7%) and IM (32.8%) groups than in the PM&R group (14.8%) (*p* < 0.001). Similarly, the rate of those who did not routinely screen for vertebral fractures was also higher in the orthopedic (38.5%) and IM (37.7%) groups (*p* < 0.001). A comparison of the diagnostic and evaluation processes by specialty is presented in Table 2.

**Table 2:** Comparison of diagnostic approaches and evaluation practices regarding osteoporosis among different medical specialties

		IM (***n*** = 122)^a^ ***n*** (%)	PM&R (***n*** = 203)^b^ ***n*** (%)	Orthopedics (***n*** = 122)^c^ ***n*** (%)	***p***-value	Cramer’s ***V***
What are your indications for ordering DXA?	Other risk factors	85 (69.7%)^c^	180 (88.7%)^a,c^	51 (41.8%)	< 0.001	0.425
	After a low-trauma fracture	87 (71.3%)	197 (97.0%)^a,c^	86 (70.5%)	< 0.001	0.382
	Patient request	52 (42.6%)	90 (44.3%)	59 (48.4%)	0.647	0.040
	I do not routinely order DXA	15 (12.3%)	12 (5.9%)	20 (16.4%)^b^	0.009	0.146
	Glucocorticoid use (> 3 months)	85 (69.7%)^c^	191 (94.1%)^a,c^	35 (28.7%)	< 0.001	0.583
	Women aged ≥ 65 years	105 (86.1%)	195 (96.1%)^a,c^	96 (78.7%)	< 0.001	0.231
	Men aged ≥ 70 years	86 (70.5%)^c^	169 (83.3%)^a,c^	47 (38.5%)	< 0.001	0.397
Which parameter do you consider most in the DXA report?	Lowest T-score	46 (37.7%)	60 (29.6%)	37 (30.3%)	0.017*	0.144
	Femoral neck T-score	39 (32.0%)	67 (33.0%)	45 (36.9%)		
	Lumbar spine T-score	27 (22.1%)	68 (33.5%)	26 (21.3%)		
	Total hip T-score	7 (5.7%)	5 (2.5%)	5 (4.1%)		
	Age-adjusted Z-score (premenopausal women and men aged < 50 years)	3 (2.5%)	3 (1.5%)	9 (7.4%)^b^		
How often do you screen for secondary causes of osteoporosis?	Never	4 (3.3%)	3 (1.5%)	26 (21.3%)^a,b^	< 0.001	0.395
	Rarely	22 (18.0%)^b^	8 (3.9%)	38 (31.1%)^b^		
	In almost every patient	3 (2.5%)	47 (23.2%)^a^	0 (0.0%)		
	If risk factors are present	93 (76.2%)^c^	145 (71.4%)^c^	58 (47.5%)		
Which Fracture Risk Assessment Tool/score do you routinely use?	FRAX	62 (50.8%)^c^	153 (75.4%)^a,c^	14 (11.5%)	< 0.001	0.528
	None	19 (15.6%)^b^	2 (1.0%)	29 (23.8%)^b^	< 0.001	0.310
	Clinical judgment only	40 (32.8%)	58 (28.6%)	78 (63.9%)^a,b^	< 0.001	0.310
How do you assess fall risk?	Simple clinical assessment	59 (48.4%)	160 (78.8%)^a,c^	63 (51.6%)	< 0.001	0.261
	I request a consultation	17 (13.9%)	0 (0.0%)	8 (6.6%)		
	I use standardized scales	6 (4.9%)	13 (6.4%)	5 (4.1%)		
	I do not assess	40 (32.8%)^b^	30 (14.8%)	46 (37.7%)^b^		
What are your indications for vertebral fracture screening?	Height loss	58 (47.5%)^c^	168 (82.8%)^a,c^	17 (13.9%)	< 0.001	0.577
	I do not perform routine screening	46 (37.7%)^b^	21 (10.3%)	47 (38.5%)^b^	< 0.001	0.319
	Glucocorticoid use	33 (27.0%)^c^	121 (59.6%)^a,c^	8 (6.6%)	< 0.001	0.481
	Back Pain	54 (44.3%)	176 (86.7%)^a,c^	58 (47.5%)	< 0.001	0.425
	T-score < −2.5	49 (40.2%)	133 (65.5%)^a,c^	47 (38.5%)	< 0.001	0.269

Cramer’s *V* was calculated for *χ*^2^ tests

A two-sided *p*-value < 0.05 was considered statistically significant

Pairwise comparisons of column proportions within the same row were performed using Bonferroni-adjusted *Z* tests; for significant differences (*p* < 0.05), the letter of the column with the smaller proportion is displayed in the column with the larger proportion. For multiple-response items, percentages were calculated based on respondents and may exceed 100%

*Monte Carlo exact *p*-values were used when > 20% of cells had expected counts < 5

*DXA*, dual-energy X-ray absorptiometry; *FRAX*, Fracture Risk Assessment Tool; *PM&R*, physical medicine and rehabilitation; *IM*, internal medicine

#### Treatment procedures and medication preferences

The most striking difference in treatment practices was the request for routine blood tests before treatment. While the percentage of those requesting routine blood tests was 99.2% in IM group and 97.5% in PM&R group, it was only 44.3% in orthopedic group (*p* < 0.001). A similar distinction was observed in the content of the blood tests. For example, the percentage of those routinely requesting kidney function tests was 95.1% in IM group and 97.0% in PM&R group, but only 39.3% in orthopedic group (*p* < 0.001). Regarding the criteria for starting treatment, the groups were similar when treatment was initiated at a T-score ≤ −2.5 (*p* = 0.069). In contrast, for all other treatment initiation criteria, the PM&R group was significantly more dominant (*p* < 0.001). In initial drug choice, oral bisphosphonate selection was higher in each group (PM&R: 92.1%; IM: 68.9%; and orthopedic: 56.6%). Intravenous zoledronate ranked second. Indications for anabolic treatment also showed significant differences among branches; for example, in the presence of “multiple fractures,” considering anabolic treatment was 66.0% in PM&R group, 20.5% in IM group, and only 6.6% in orthopedic group (*p* < 0.001). In situations where denosumab would be preferred, especially in cases where glomerular filtration rate (GFR) < 30 mL/min, the preference for denosumab was 92.1% in PM&R group, 62.3% in IM group, and only 15.6% in orthopedic group (*p* < 0.001). Additionally, the response “I do not use denosumab” was 40.2% in orthopedic group, while it was 0.0% in PM&R group (*p* < 0.001). No significant difference was observed among the groups in terms of providing written lifestyle recommendations (*p* = 0.081). A comparison of treatment practices and drug preferences according to specialties is presented in Table 3.

**Table 3:** Comparison of osteoporosis treatment practices and medication preferences among different medical specialties

		IM (*n* = 122)^a^	PM&R (*n* = 203)^b^	Orthopedics (*n* = 122)^c^	*p*-value	Cramer’s ***V***
		*n* (%)	*n* (%)	*n* (%)		
What are your criteria for initiating treatment?	After a low-trauma fracture	56 (45.9%)	168 (82.8%)^a,c^	55 (45.1%)	< 0.001	0.383
	FRAX major osteoporotic fracture risk ≥ 20% or hip fracture risk ≥ 3%	52 (42.6%)	141 (69.5%)^a,c^	34 (27.9%)	< 0.001	0.357
	T-score ≤ −2.5	108 (88.5%)	189 (93.1%)	104 (85.2%)	0.069	0.109
	High fall risk	13 (10.7%)	57 (28.1%)^a,c^	13 (10.7%)	< 0.001	0.223
Do you routinely order blood tests before initiating osteoporosis treatment?	Yes	121 (99.2%)^c^	198 (97.5%)^c^	54 (44.3%)	< 0.001	0.646
	No	1 (0.8%)	5 (2.5%)	68 (55.7%)^a,b^		
If you order blood tests, which parameters do you consider essential?	Vitamin D	12 (9.8%)	37 (18.2%)^c^	2 (1.6%)	< 0.001	0.218
	Renal function tests	116 (95.1%)^c^	197 (97.0%)^c^	48 (39.3%)	< 0.001	0.644
	Complete blood count	94 (77.0%)	163 (80.3%)^c^	47 (38.5%)	< 0.001	0.388
	Liver function tests	83 (68.0%)	170 (83.7%)^a,c^	45 (36.9%)	< 0.001	0.411
	Bone turnover markers	55 (45.1%)^b^	42 (20.7%)	34 (27.9%)^b^	< 0.001	0.222
	Electrolytes	121 (99.2%)^c^	198 (97.5%)^c^	62 (50.8%)	< 0.001	0.595
	Parathyroid hormone	89 (73.0%)^c^	174 (85.7%)^a,c^	35 (28.7%)	< 0.001	0.506
Do you prescribe vitamin D and calcium replacement before treatment?	I prescribe it for all patients	72 (59.0%)^b^	79 (38.9%)	57 (46.7%)	< 0.001	0.152
	Only in patients with deficiency	50 (41.0%)	124 (61.1%)^a^	62 (50.8%)		
	I do not recommend it	0 (0.0%)	0 (0.0%)	3 (2.5%)		
What is your most common first-line drug preference?	Oral bisphosphonate	84 (68.9%)	187 (92.1%)^a,c^	69 (56.6%)	< 0.001	0.298
	Intravenous zoledronate	30 (24.6%)^b^	7 (3.4%)	30 (24.6%)^b^		
	Others	4 (3.3%)	9 (4.4%)	6 (4.9%)		
	No preference	4 (3.3%)	0 (0.0%)	17 (13.9%)^a^		
Do you prescribe pharmacologic treatment in osteopenia?	Yes, if FRAX risk is high	48 (39.3%)	92 (45.3%)^c^	33 (27.0%)	0.005	0.155
	Yes, if a fracture is present	38 (31.1%)	140 (69.0%)^a,c^	41 (33.6%)	< 0.001	0.364
	Depends on the clinical scenario	47 (38.5%)	76 (37.4%)	38 (31.1%)	0.414	0.063
	Generally no	22 (18.0%)^b^	12 (5.9%)	24 (19.7%)^b^	< 0.001	0.193
Do you recommend pharmacologic treatment for osteopenic patients who will receive glucocorticoids?	Never	0 (0.0%)	0 (0.0%)	0 (0.0%)	< 0.001	0.278
	Rarely	18 (14.8%)	33 (16.3%)	53 (43.4%)^a,b^		
	Sometimes	14 (11.5%)	47 (23.2%)^a^	31 (25.4%)^a^		
	Often	52 (42.6%)^c^	94 (46.3%)^c^	30 (24.6%)		
	Always	38 (31.1%)^b,c^	29 (14.3%)	8 (6.6%)		
Do you consider whether the medication is effective across all fracture types?	Never	8 (6.6%) b	3 (1.5%)	27 (22.1%)^a,b^	< 0.001	0.392
	Rarely	19 (15.6%)^b^	5 (2.5%)	33 (27.0%)^b^		
	Sometimes	25 (20.5%)^b^	16 (7.9%)	27 (22.1%)^b^		
	Often	43 (35.2%)^c^	91 (44.8%)^c^	24 (19.7%)		
	Always	27 (22.1%)^c^	88 (43.3%)^a,c^	11 (9.0%)		
In which situations do you prescribe anabolic therapy?	Inadequate response to bisphosphonates	64 (52.5%)^c^	91 (44.8%)^c^	28 (23.0%)	< 0.001	0.233
	Renal insufficiency	36 (29.5%)^c^	63 (31.0%)^c^	11 (9.0%)	< 0.001	0.246
	I do not use anabolic therapy	36 (29.5%)^b^	16 (7.9%)	50 (41.0%)^b^	< 0.001	0.338
	Very low T-score	24 (19.7%)	116 (57.1%)^a,c^	28 (23.0%)	< 0.001	0.369
	Very high fracture risk	23 (18.9%)	107 (52.7%)^a,c^	29 (23.8%)	< 0.001	0.329
	Multiple fractures	25 (20.5%)^c^	134 (66.0%)^a,c^	8 (6.6%)	< 0.001	0.551
In which situations do you prefer denosumab?	Bisphosphonate intolerance/nonadherence	63 (51.6%)^c^	192 (94.6%)^a,c^	36 (29.5%)	< 0.001	0.59
	Inadequate response to bisphosphonates	69 (56.6%)^c^	188 (92.6%)^a,c^	36 (29.5%)	< 0.001	0.56
	First-line choice	2 (1.6%)	3 (1.5%)	4 (3.3%)	0.577	0.055
	I do not use denosumab	25 (20.5%)	0 (0.0%)	49 (40.2%)^a^	< 0.001	0.451
	Renal impairment (GFR < 30 mL/min/1.73 m^2^)	76 (62.3%)^c^	187 (92.1%)^a,c^	19 (15.6%)	< 0.001	0.655
Do you provide written lifestyle recommendations (exercise, fall prevention, etc.)?	Never	18 (14.8%)^b^	13 (6.4%)	19 (15.6%)^b^	0.081	0.125
	Rarely	13 (10.7%)	22 (10.8%)	18 (14.8%)		
	Sometimes	20 (16.4%)	24 (11.8%)	18 (14.8%)		
	Often	29 (23.8%)	61 (30.0%)	26 (21.3%)		
	Always	42 (34.4%)	83 (40.9%)	41 (33.6%)		

Cramer’s *V* was calculated for *χ*^2^ tests

A two-sided *p*-value < 0.05 was considered statistically significant

Pairwise comparisons of column proportions within the same row were performed using Bonferroni-adjusted *Z* tests; for significant differences (*p* < 0.05), the letter of the column with the smaller proportion is displayed in the column with the larger proportion. For multiple-response items, percentages were calculated based on respondents and may exceed 100%

*GFR*, glomerular filtration rate; *FRAX*, Fracture Risk Assessment Tool; *PM&R*, physical medicine and rehabilitation; *IM*, internal medicine

#### Follow-up and monitoring strategies

The most preferred DXA parameter used in follow-up was the T-score. However, among those who used bone mineral density in follow-up, the rate was 45.8% in PM&R group, 34.4% in IM group, and only 3.3% in orthopedic group (*p* < 0.001). There were also differences between the groups in the approach to drug holidays during oral bisphosphonate use (*p* < 0.001). For example, the proportion of those considering a drug holiday by re-evaluating the patient after 3–5 years and if the fracture risk is low was 92.1% in PM&R group, 69.7% in IM group, and 50.0% in orthopedic group. In the follow-up of height loss, the response “I never do it” was higher in orthopedic group, at 52.5% (*p* < 0.001). The application of biochemical monitoring in patients under treatment also differed between the groups (*p* < 0.001). The response “I do not monitor” was 23.8% in orthopedic group, while it was 2.5% in IM group and 3.0% in PM&R group. For stable patients not receiving treatment, the interval for repeating DXA in all groups was predominantly “1 year.” There was a marked divergence between groups in planning transition therapy after discontinuation of denosumab (*p* < 0.001). The answer “Yes, always” was 80.3% in PM&R group, 32.0% in IM group, and 12.3% in orthopedic group. In contrast, the response “I do not use denosumab” was 53.3% in orthopedic group and 33.6% in IM group, but only 1.0% in PM&R group. There were also differences between groups regarding the question of who should be the primary responsible physician in osteoporosis management (*p* < 0.001). The proportion of those who stated that PM&R physicians should be primarily responsible was 89.7% in the PM&R group. In contrast, the proportion of those who said IM-endocrinology specialists should be primarily responsible was 54.9% in IM group, 44.3% in orthopedic group, and only 6.9% in PM&R group. A comparison of follow-up and monitoring practices by specialty is presented in Table 4.

**Table 4:** Comparison of osteoporosis follow-up and long-term monitoring strategies among different medical specialties

		IM (*n* = 122)^a^ *n* (%)	PM&R (*n* = 203)^b^ *n* (%)	Orthopedics (*n* = 122)^c^ *n* (%)	***p***-value	Cramer’s ***V***
Which DXA parameters do you use during follow-up?	All parameters	27 (22.1%)^c^	43 (21.2%)^c^	10 (8.2%)	0.005	0.155
	Bone mineral density	42 (34.4%)^c^	93 (45.8%)^c^	4 (3.3%)	< 0.001	0.382
	T-score	80 (65.6%)	157 (77.3%)	100 (82.0%)^a^	0.008	0.146
	Z-score	22 (18.0%)	34 (16.7%)	23 (18.9%)	0.884	0.023
What is your approach to a “drug holiday” with oral bisphosphonates?	I do not recommend a drug holiday	7 (5.7%)	0 (0.0%)	12 (9.8%)	< 0.001	0.29
	Depends on the clinical scenario	30 (24.6%)^b^	16 (7.9%)	49 (40.2%)^a,b^		
	I reassess after 3–5 years and consider a drug holiday if fracture risk is low	85 (69.7%)^c^	187 (92.1%)^a,c^	61 (50.0%)		
Do you perform annual height measurements or ask patients about height loss?	Never	28 (23.0%)	32 (15.8%)	64 (52.5%)^a,b^	< 0.001	0.334
	Rarely	47 (38.5%)^b^	35 (17.2%)	33 (27.0%)		
	Sometimes	26 (21.3%)	51 (25.1%)^c^	16 (13.1%)		
	Often	15 (12.3%)	50 (24.6%)^a,c^	8 (6.6%)		
	Always	6 (4.9%)	35 (17.2%)^a,c^	1 (0.8%)		
Do you perform biochemical monitoring in patients receiving treatment? If yes, how often?	I do not perform it	3 (2.5%)	6 (3.0%)	29 (23.8%)^a,b^	< 0.001	0.341
	Only if symptoms are present	4 (3.3%)	17 (8.4%)	31 (25.4%)^a,b^		
	At 3–6-month intervals	84 (68.9%)^b,c^	103 (50.7%)^c^	32 (26.2%)		
	Annually	31 (25.4%)	77 (37.9%)^c^	30 (24.6%)		
What is your interval for repeat DXA in stable/untreated patients?	1 year	57 (49.1%)	81 (40.1%)	81 (70.4%)^a,b^	< 0.001**	0.196
	2 years	38 (32.8%)^c^	60 (29.7%)^c^	16 (13.9%)		
	3 years	4 (3.4%)	6 (3.0%)	4 (3.5%)		
	Depends on the clinical scenario	17 (14.7%)	55 (27.2%)^a,c^	14 (12.2%)		
Do you plan transition therapy after discontinuation of denosumab?	Yes, always	39 (32.0%)^c^	163 (80.3%)^a,c^	15 (12.3%)	< 0.001	0.455
	Sometimes	32 (26.2%)^b^	30 (14.8%)	31 (25.4%)		
	No	10 (8.2%)	8 (3.9%)	11 (9.0%)		
	I do not use denosumab	41 (33.6%)^b^	2 (1.0%)	65 (53.3%)^a,b^		
Do you request an endocrinology consultation when you encounter a problem during follow-up?	Never	4 (3.3%)	7 (3.4%)	12 (9.8%)	< 0.001	0.244
	Rarely	18 (14.8%)	37 (18.2%)	20 (16.4%)		
	Sometimes	29 (23.8%)	86 (42.4%)^a,c^	26 (21.3%)		
	Often	33 (27.0%)	61 (30.0%)	34 (27.9%)		
	Always	38 (31.1%)^b^	12 (5.9%)	30 (24.6%)^b^		
Who should be the primary clinician responsible for osteoporosis management?	Internal Medicine-Endocrinology	67 (54.9%)^b^	14 (6.9%)	54 (44.3%) ^b^	< 0.001*	0.391
	PM&R	47 (38.5%)	182 (89.7%)^a,c^	48 (39.3%)		
	Orthopedics	2 (1.6%)	0 (0.0%)	9 (7.4%)^a^		
	Primary Care Physician	6 (4.9%)	7 (3.4%)	11 (9.0%)		

Cramer’s *V* was calculated for *χ*^2^ tests

A two-sided *p*-value < 0.05 was considered statistically significant

Pairwise comparisons of column proportions within the same row were performed using Bonferroni-adjusted *Z* tests; for significant differences (*p* < 0.05), the letter of the column with the smaller proportion is displayed in the column with the larger proportion. For multiple-response items, percentages were calculated based on respondents and may exceed 100%

*Monte Carlo exact *p*-values were used when > 20% of cells had expected counts < 5

**“I don’t perform DXA” responses were excluded (*n* = 14); analysis performed on *n* = 433

*PM&R*, physical medicine and rehabilitation; *IM*, internal medicine; *DXA*, dual-energy X-ray absorptiometry

### Awareness questions

The osteoporosis awareness score, created from the awareness-related questions in the survey, differed significantly between groups (*p* < 0.001). The highest median (IQR) score was observed in the PM&R group at 4.00 (3.75–4.25), followed by the IM group at 3.50 (3.00–4.00), and the orthopedic group at 2.88 (2.00–3.50). The linear regression model established for the awareness score was significant (*R*^2^ = 0.383; adj *R*^2^ = 0.375; *F* = 45.546; *p* < 0.001). Multiple linear correlations were examined using tolerance and VIF values, and no significant multicollinearity was detected (tolerance: 0.516–0.766; VIF: 1.305–1.938). When the orthopedic group was taken as the reference, being from the PM&R (*B* = 0.782; *β* = 0.435; *p* < 0.001) and IM (*B* = 0.509; *β* = 0.253; *p* < 0.001) specialties were independently associated with a higher awareness score. Additionally, the use of guidelines was one of the independent positive determinants of the awareness score (*B* = 0.344; *β* = 0.350; *p* < 0.001). Total experience, academic title, and monthly patient volume did not independently contribute to the model (all *p* > 0.05). The multivariable regression analysis model established for the awareness score is provided in Table 5.

**Table 5:** Multivariable linear regression analysis of factors associated with awareness score

Variable	Unstandardized *B*	Std. error	Standardized *β*	*t*	*p*-value	95% CI for *B*	VIF
(Constant)	1.998	0.127	-	15.773	< 0.001	[1.749, 2.246]	
Specialty (Ref: orthopedics)							
- PM&R	0.782	0.093	0.435	8.393	< 0.001	[0.599, 0.965]	1.915
- IM	0.509	0.093	0.253	5.487	< 0.001	[0.327, 0.691]	1.517
Guideline use	0.344	0.043	0.350	7.917	< 0.001	[0.259, 0.429]	1.390
Total experience	0.052	0.033	0.079	1.554	0.121	[−0.014, 0.117]	1.831
Academic rank	0.028	0.044	0.033	0.639	0.523	[−0.058, 0.114]	1.938
Monthly patient volume	−0.001	0.027	−0.002	−0.046	0.963	[−0.055, 0.052]	1.305

*R*^2^ = 0.383; adjusted *R*^2^ = 0.375; *F* = 45.546, *p* < 0.001. Ordinal predictors were entered as ordered numeric variables. Dummy variables used for specialty with orthopedics as the reference category

*PM&R*, physical medicine and rehabilitation; *IM*, internal medicine; *CI*, confidence interval

## Discussion

The main finding of this study is that practices related to the diagnosis, treatment, and follow-up steps of osteoporosis differ significantly among PM&R, IM, and orthopedic physicians. When the survey data were evaluated, PM&R physicians demonstrated the highest compliance with national and international guidelines. In contrast, it was observed that orthopedic physicians showed lower concordance with guideline-based care. Overall, IM physicians occupied an intermediate position between the two groups. The high internal consistency of the awareness scale (*α* = 0.890) supports the reliability of the composite measure; the observed between-specialty differences may reflect differences in training exposure and clinical focus. Another important finding was that the proportion of physicians in each specialty who either did not have an FLS in their institution or were unaware of it was high.

Risk assessment tools have been developed to evaluate the likelihood of future fractures in patients with osteoporosis. Among these, the FRAX Fracture Risk Assessment Tool, developed in 2008 by the World Health Organization Collaborating Centre for Metabolic Bone Diseases at the University of Sheffield, is the most popular and widely used [15]. The use of FRAX in clinical practice is strongly recommended by both international and national guidelines [1, 12, 15]. In our study, the most notable distinction in the diagnostic process was the use of FRAX Fracture Risk Assessment Tools. While the routine use of FRAX in the clinic was 11.5% among orthopedic physicians, it was found to be 50.8% among IM physicians and 75.4% among PM&R physicians. Our findings are consistent with previous studies, such as one conducted by Aydın among physicians treating osteoporosis in Türkiye, where only 13.3% of physicians reported using the FRAX fracture risk tool, and another by Bakılan et al. reporting FRAX awareness among orthopedic physicians as only 17.2% [11, 16]. These findings suggest that the primary focus of surgical specialties may be the detection and treatment of fractures rather than the diagnosis and treatment of osteoporosis itself. Similarly, low guideline adherence rates for vertebral fracture screening and DXA ordering indications further indicate that secondary prevention has not yet become a primary clinical priority in orthopedic practice.

In the literature, the proportion of patients suitable for the pharmacological treatment of osteoporosis but are not treated is defined as the treatment gap [17]. Additionally, definitions have been made in terms of the proportion of patients at increased risk of fragility who do not receive treatment or the proportion of patients who do not receive treatment for a certain period (6 months) after a low-trauma fracture [18, 19]. In Europe, the treatment gap has been reported to be as high as 90% [18, 19]. Numerous factors contribute to this treatment gap, such as ambiguity regarding primary responsibility, lack of FLS and coordination, physicians’ non-adherence to guidelines, and time constraints in outpatient clinics [8, 17]. In a study conducted by Çakır et al. at a tertiary center in Türkiye, 83.1% of patients did not receive any osteoporosis treatment after a hip fracture, and mortality was significantly higher in this untreated group [20]. Similarly, in a study conducted by Aydın on orthopedic physicians, 63.9% reported not assessing osteoporosis in elderly patients after a fracture [16]. In the current study, the finding that only 45.1% of orthopedic physicians initiated treatment after a low-trauma fracture may reflect an important treatment gap.

In our study, there were differing attitudes regarding which specialty should bear the primary responsibility for osteoporosis management. While 89.7% of PM&R physicians believed they should be the primary responsible party, only 7.4% of orthopedic physicians thought the same for themselves, with 44.3% stating that IM physicians should hold primary responsibility. Additionally, among orthopedic physicians, 97.5% either did not have a FLS at their institution or were unaware of its existence. Furthermore, orthopedic physicians scored significantly lower than other specialties on the guideline awareness score derived from our survey questions. Taken together, these findings, confusion over primary responsibility, the absence or unawareness of FLS, and the low awareness among orthopedic physicians who intervene in osteoporotic fractures can be considered factors contributing to the treatment gap, in line with the literature.

In our study, while the rate of routine pre-treatment laboratory screening among orthopedic physicians remained at 44.3%, it increased to 99.2% among IM physicians and 97.5% among PM&R physicians. This situation may demonstrate that essential laboratory tests such as vitamin D, calcium, and GFR monitoring for maintaining metabolic balance in osteoporosis management, as recommended in the consensus report published by Lei et al. and guidelines, are neglected in surgical practice [1, 12, 21]. This situation may also result in an increased risk of overlooking secondary causes of osteoporosis. Furthermore, the low rates of preference for anabolic agents and denosumab show that orthopedic physicians still maintain a traditional and limited approach to the medical treatment of osteoporosis.

Our study revealed a significant gap in the management of transition therapy following denosumab. While 80.3% of PM&R physicians plan transition therapy after denosumab, this rate remains at 32.0% in the IM physicians and only 12.3% in the orthopedic physicians, indicating a lack of knowledge regarding the need for sequential therapy as specified in guidelines and some publications [1, 12, 22]. These interdepartmental differences in the medical follow-up of patients may lead to an increased risk of refracture in the near future.

The regression analysis conducted for the awareness score we created revealed that academic seniority and years of professional experience were not independent determinants. In contrast, active guideline usage and specialty were strong independent determinants. This result parallels the findings of Mahdaviazad et al. and Hawarden et al., who emphasized the importance of continuing medical education and guideline familiarity [8, 23]. In other words, osteoporosis management is shaped not only by experience and seniority but also by the active following of current guidelines and the emphasis placed on them in specialty training programs.

Ülgü et al., in their study based on the national health database, reported that there are 4.2 million osteoporosis patients in Türkiye, thereby highlighting the scale of the burden [24]. Given this high prevalence and treatment gap, missed opportunities in surgical units and the lack of familiarity with FLS continue to be serious systemic issues. As a solution, the widespread implementation of FLS, which has been shown to reduce mortality and morbidity, and improved coordination are of vital importance for the management of osteoporosis patients [1, 6].

Our findings indicate that osteoporosis should no longer be considered a disease with unclear status, caught in the confusion of interdepartmental responsibilities. Without coordinated efforts between orthopedic physicians in managing osteoporotic fractures, the medical treatment approaches of PM&R and IM physicians, and the operation of the FLS, it will not be possible to cope with the ever-increasing epidemiological burden of osteoporosis.

This study has several limitations. First, because the data were collected through a self-reported online survey, the responses may not fully reflect actual clinical practice and may have been influenced by social desirability bias. Second, because the survey was distributed through professional association messaging groups and digital networks, the total number of physicians who received the invitation could not be determined; therefore, the response rate could not be calculated and the sample should be considered a convenience sample. Third, although responses were reviewed for completeness and consistency, duplicate entries or participant interaction could not be completely excluded because of the anonymous online design. Fourth, the cross-sectional design precludes causal inference. Finally, only three specialties were included, which may limit the generalizability of the findings. Despite these limitations, this study represents one of the largest inter-specialty comparative surveys on osteoporosis guideline adherence and awareness conducted in Türkiye. In addition, the relatively large sample size and the use of multivariable regression analysis strengthen the robustness of the findings.

## Conclusion

In Türkiye, osteoporosis management exhibits significant differences in approach among various specialties. While PM&R physicians show high adherence to guidelines, physicians in orthopedics and traumatology appear less concordant with guideline-based medical management, which may partly relate to the fracture-focused nature of their clinical practice. To address treatment gaps and cope with the increasing epidemiological burden of osteoporosis, osteoporosis education and active guideline use should be restructured and emphasized during residency training. Additionally, the active integration of FLS into the system appears to be necessary.

## Data Availability

The datasets generated and analyzed during the current study are not publicly available due to ethical and confidentiality considerations but are available from the corresponding author upon reasonable request.
